# Immunomodulatory effects of iTr35 cell subpopulation and its research progress

**DOI:** 10.1007/s10238-024-01303-5

**Published:** 2024-02-22

**Authors:** Chenxi Yang, Lingli Dong, Jixin Zhong

**Affiliations:** 1 Department of Rheumatology and Immunology, Tongji Hospital, Huazhong University of Science and Technology, Wuhan, 430030 Hubei China; 2 Institute of Allergy and Clinical Immunology, Tongji Hospital, Tongji Medical College, Huazhong University of Science and Technology, Wuhan, 430030 Hubei China

**Keywords:** Induced regulatory T cells 35, Interleukin 35, Immunomodulation

## Abstract

The spotlight in recent years has increasingly focused on inducible regulatory T cells 35 (iTr35), a novel subpopulation of regulatory T cells characterized by phenotypic stability, heightened reactivity, and potent immunosuppressive function through the production of IL-35. Despite being in the exploratory phase, research on iTr35 has garnered significant interest. In this review, we aim to consolidate our understanding of the biological characteristics and immunomodulatory mechanisms of iTr35, offering fresh perspectives that may pave the way for its potential applications in disease diagnosis and treatment.

## Introduction

**Fig. 1:**
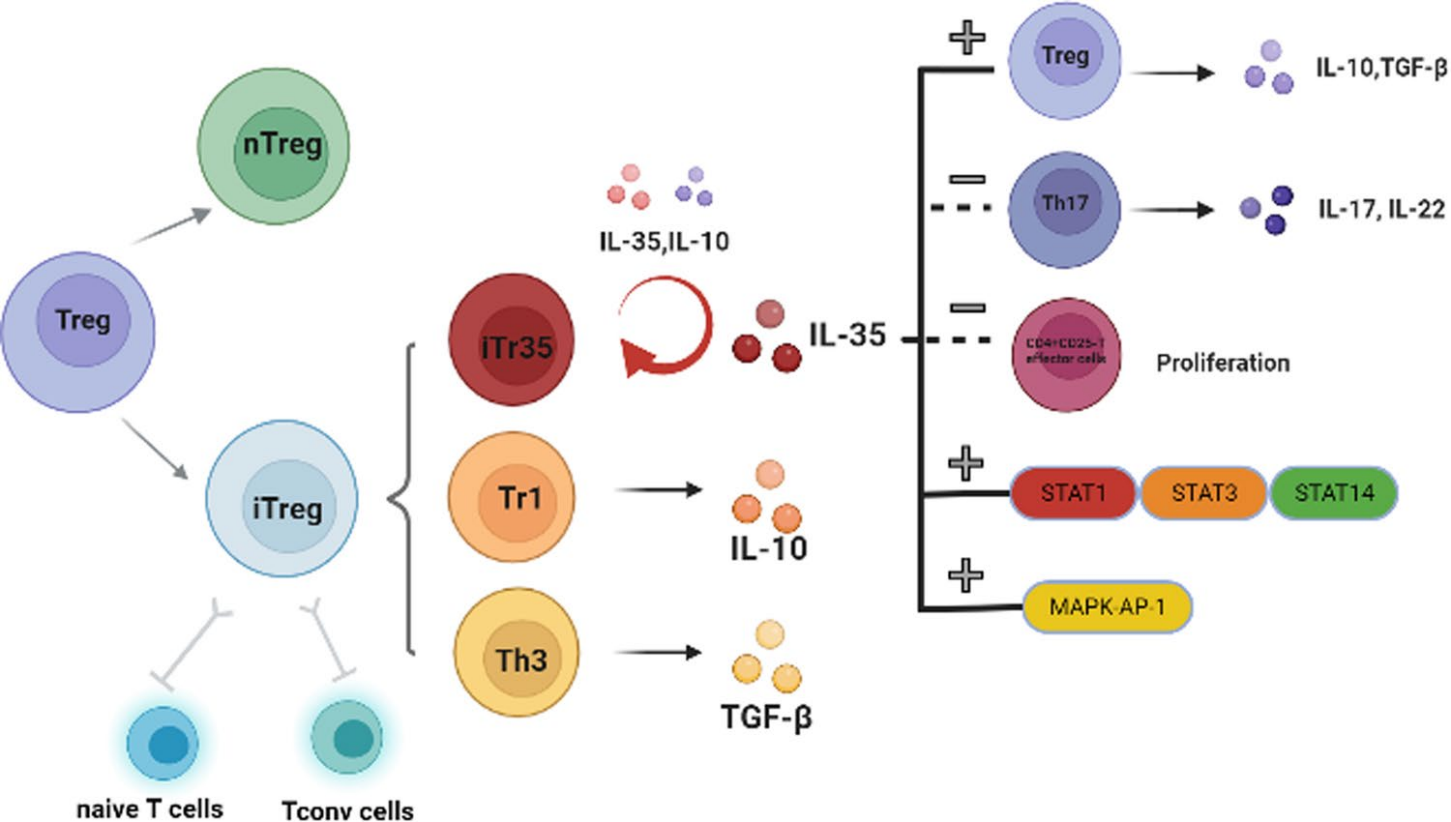
Biological characteristics and immunomodulatory mechanisms of iTr35.Treg: Regulatory T cell; nTregs: Naturally occurring Tregs; iTregs: Induced Tregs; Tconv cells: Conventional T cells; iTr35: Inducible regulatory T cells 35;Tr1: Inducible regulatory T cells 1

Regulatory T cells (Tregs) play a crucial role in maintaining immune tolerance by suppressing excessive immune response [1]. Two subsets of Tregs have been confirmed: naturally occurring Tregs (nTregs) that occur and develop in the thymus and induced Tregs (iTregs) that are generated from natural conventional T cells (Tconv) in the periphery. Three types of iTreg cells have been further categorized based on the cytokines produced by these cells: iTr-TGF-β, iTr-IL-10, and iTr35 cells. Among these subsets, iTr35 (inducible regulatory T cells35) is a newly identified functional subpopulation of iTregs that are phenotypically stable and suppressive. iTr35 does not express forkhead box protein P3 (Foxp3), the key transcription factor for Treg, while it highly expresses p35 and EBI3 and exerts potent immunosuppressive function through secretion of mature interleukin-35 (IL-35) [2, 3] (Fig. 1). The IL-35 receptor consists of IL-12Rβ2 and gp130. The homodimeric receptor alone does not promote iTr35 cell differentiation. However, it co-transduced through both signal transduction transcriptional activator 1 (STAT1) and 4 (STAT4) pathways, which induce iTr35 cell production and form a positive feedback loop to produce more iTr35 [4]. iTr35 has a promising potential in clinical therapy as it is stably circulating in the human body. However, the specific physiological mechanisms underlying the stability of iTr35 in vivo remain unknown. The research on iTr35 is still in the exploratory stage and has been mainly studied in animals in a few autoimmune diseases. In Foxp3-/- mice with experimental colitis, iTr35 cells, like nTreg cells, restored the homeostasis of the immune system, suppressed T cell proliferation, and prevented the development of colitis [5]. In inflammatory diseases, iTr35 cells may lose their regulatory function and sometimes acquire the phenotypic characteristics of effector cells [6]. However, it has also been shown that T cells in various tumors are rich in IL-35 cytokines and that the transfer of iTr35 cells promotes tumor development [7]. Recently, the study of iTr35 cells in inflammatory and fibrotic lesions in systemic sclerosis has been explored, providing a reference for the treatment of autoimmune diseases with iTr35 [8, 9]. In this review, we explore the role of iTr35, especially its biological characteristics and immunomodulatory mechanism in the pathogenesis of diseases, aiming to provide new ideas for the treatment of diseases.

## Discovery of iTr35 cells

Treg cells have many inhibitory mechanisms, including the release of suppressive cytokines such as transforming growth factor-β (TGF-β) and interleukin-10 (IL-10), as well as regulating cell maturation or functional mechanisms, such as cytotoxic T lymphocyte-associated protein 4 ( CTLA4) expression [10, 11]. To date, there are two major classes of Treg cells: naturally occurring Treg cells (natural Treg, nTreg) that originate from the thymus and migrate into the periphery, which are highly expressed with the transcription factor Foxp3 and have immunomodulatory and immunosuppressive functions, and induced Treg cells (induced Treg, iTreg) that are generated in the peripheral environment through different mechanisms. The induced Treg (iTreg) is differentiated from naive T cell (Tnaive) in the peripheral environment through different mechanisms, including Tr1 (Foxp3-, secreting IL-10) and Th3 (Foxp3-, secreting TGF-β) [12]. These iTreg are mainly produced in response to specific antigens or cytokines [13]. In 2010, Collison et al. [7]. found that IL-35 was able to induce human or mouse CD4 + T cells to produce a novel subpopulation of iTreg cells with a long-term stable phenotype and suppressive function, and named them iTr35 cells.

## Biological functions of iTr35 cells

The subsequent studies confirmed that iTr35 cells exert immunosuppressive effects by secreting IL-35, independent of Foxp3 and classical suppressive cytokines IL-10 and TGF-β [7]. IL-35 induced high expressions of Epstein-Barr virus-induced gene 3 (EBI3) and P35 in human naive T cells, whereas the expressions of these genes in naive cells were unchanged by IL-10 and TGF-β. In addition, the naive T cells pretreated with IL-35 were co-cultured with Teff cells in a Transwell® chamber and the pretreated cells could inhibit the proliferation of effector T cells (Teff) in a non-contact manner, suggesting that iTr35 could play a regulatory role by secreting the immunosuppressive cytokine IL-35.

## Immunomodulatory effects of iTr35 cells (Table 1)

**Table 1:** Immunomodulatory effects of iTR35 cells

Diseases	Mechanisms
Mycobacterium tuberculosis infection	Increased bacterial load and lung injury
Allergic rhinitis	Inhibited the proallergic type 2 immune responses by reducing ILC2 cytokines IL-5 and IL-13 and Th2 cytokines IL-4, IL-5, IL-9, and IL-13; inhibited CD40L, IL-4, and IL-21 stimulated B-cell IgE production
Allergic asthma	Suppress the proliferation and IL-4 production by activated CD4 + CD25- T cells
Oral lichen planus	Increased percentage of iTr35 subpopulation
Acute phase Kawasaki disease	iTr35 significantly reduced
Systemic sclerosis	Inhibited T cell proliferation and differentiation and fibroblast-induced α-SMA expression through the STAT3/6 signaling pathway
Maternal–fetal tolerance	Reduction in the number of metaphase iTr35 is also important cause of spontaneous abortion
Myelodysplastic syndromes	iTr35 cells increased significantly with the increase of disease risk classification
Acute B-precursor lymphocytic leukemia	Immune escape process of tumor cells
Acute myeloid leukemia	Highly expressed, significantly correlated with the clinical stage of malignancy
Colorectal cancer	Inhibited the proliferation of effector T cells, activated STAT1 and STAT3 CD4 + T cells
Breast cancer	Induced iTr35 by activating the transcription factors STAT1/STAT3
Atherosclerosis	Immunosuppressive and anti-inflammatory activity
Fibrotic diseases	Inhibit fibrosis

Instead of expressing Foxp3, iTr35 cells overexpress P35 and EBI3. They also specifically secrete IL-35, which is closely associated with infectious diseases, autoimmune diseases, tumors, and inflammation [7].

### iTr35 cells and mycobacterium tuberculosis infection

Mycobacterium tuberculosis infection that causes human tuberculosis remains a worldwide public health problem. As an effective vaccine has not been fully developed, researchers have investigated the mechanisms of host–pathogen interactions and immune escape mediated by chronic infection of Mycobacterium tuberculosis. Cellular immunity-mediated protection against Mycobacterium tuberculosis can serve as a biomarker of bacterial infection [14], where Treg and regulatory B cells (Regulatory B cells, Bregs) have a key role as an immune negative regulator, mainly because they secrete high levels of IL-10, TGF-β, and IL-35. Studies on the role of IL-35 and iTr35 cells in chronic infectious tuberculosis are limited. Yu et al. [15] observed that mice infected with Mycobacterium tuberculosis resulted in elevated IL-35 and iTr35 subpopulations and increased bacterial load and lung injury, suggesting that IL-35 and iTr35 cells may play an immunosuppressive role in chronic Mycobacterium tuberculosis-infected mice, which provides new evidence for the treatment potential of iTr35 blockade in tuberculosis.

### iTr35 cells and allergic rhinitis

Allergic rhinitis is a major health epidemic affecting up to 40% of the global population [16], and allergen immunotherapy is a therapeutic approach for disease remission. Studies have found that IL-35 production by iTr35 cells inhibited the proallergic type 2 immune responses by reducing ILC2 cytokines IL-5 and IL-13 and Th2 cytokines IL-4, IL-5, IL-9, and IL-13, in patients with grass pollen allergy. Most importantly, IL-35 inhibited CD40L, IL-4, and IL-21 stimulated B-cell IgE production, identifying that IL-35 and iTr35 cells are important immunomodulators induced by sublingual immunotherapy, providing a theoretical basis for IL-35 therapy in the treatment of respiratory allergic diseases [17, 18]. Recently, iTr35 has also been found to inhibit ILC2s through ICOS: ICOSL-mediated cell–cell contact and IL-35 production [19].

### iTr35 cells and allergic asthma

Allergic asthma is a chronic airway inflammation resulting from an imbalanced T helper (Th) cell response to allergens. IL-35 effectively suppresses the proliferation and IL-4 production by activating CD4 + CD25- T cells (Teff cells) and the IL-35 levels are reduced in asthma patients [20]. However, the cause for the reduction of IL-35 in allergic asthma patients is unclear. Wang et al. [21] found that the ability to produce iTr35 cells was significantly reduced in asthma patients. iTr35 cells inhibited allergen-driven differentiation of naive CD4 + T cells to Th2 cells, Teff cell proliferation, and Th2 cytokine production in an IL-35-dependent manner. Therefore, iTr35 cells may be a potential immune mediator with anti-allergic effects and the potential to promote allergen tolerance.

### iTr35 cells and oral lichen planus

Oral lichen planus is a T cell-mediated chronic inflammatory disease of the oral mucosa, and Treg cells are thought to be involved in its pathogenesis [22]. Study found a serum level of elevated IL-35 and an increased percentage of iTr35 subpopulation in patients with oral lichen planus, when compared to healthy controls, suggesting that IL-35 and iTr35 cells may be involved in the pathogenesis of oral lichen planus [23].

### iTr35 cells and acute phase Kawasaki disease

The etiology and pathogenesis of Kawasaki disease are unclear. Currently, Kawasaki disease is considered an autoimmune vasculitis syndrome caused by infection [24], but the specific immunomodulatory mechanisms have not been clarified. The research have found that iTr35 and IL-35 were significantly reduced in the peripheral blood of patients with acute-phase Kawasaki disease, while the number of iTr35 cells recovered significantly after treatment. It is hypothesized that the reduced level and abnormal function of iTr35 cells may be one of the key factors leading to the abnormal activation of the immune system in the acute phase of Kawasaki disease, providing a direction for the development of new treatments for Kawasaki disease [25].

### iTr35 cells and systemic sclerosis

Systemic sclerosis is an autoimmune disease characterized by skin and organ fibrosis and vascular disease. The pathogenesis of SSc is not fully understood and the role of IL-35 in the pathogenesis of SSc remains controversial. Dantas et al. [26] first reported that elevated serum IL-35 levels in SSc patients were positively correlated with pulmonary fibrosis and could be used as a serological marker for SSc. Subsequently, extensive studies have also shown elevated serum IL-35 levels in SSc patients [2, 27], negatively correlated with disease duration [2], disease severity, and modified Rodnan skin score (mRSS) [26]. Recent studies have found reduced levels of Th1/Th2 but elevated levels of Tregs and IL-35 in the blood of SSc patients when compared to healthy controls. rhIL-35 treated suppressed the proliferation of human skin fibroblasts with CD4 + T lymphocytes in patients [8]. Further studies revealed that iTr35 and Tr1 are key T cell subsets that regulate the inflammatory response and fibrotic lesions in the pathogenesis of SSc. IL-35 promotes the production of iTr35 cells via IL-35- and IL-10-dependent mechanisms [9]. In addition, iTr35 cells inhibited the proliferation and differentiation of T cells and fibroblast-induced α-SMA expression through STAT3/6 signaling pathways [9]

### iTr35 cells and maternal–fetal tolerance

The fetus is considered a homozygous graft present in the immunocompetent mother, and the maternal immune system must tolerate this homozygous fetus to maintain the pregnancy [28]. The maintenance of maternal–fetal tolerance is achieved through the synergistic action of cells and cytokines in the maternal–fetal interface [29, 30]. However, the mechanisms behind this unique immune behavior remain poorly understood. As a key component of the human placenta, trophoblasts, in addition to their proliferative and invasive properties, express various chemokines and cytokines to maintain maternal–fetal tolerance [31]. Liu et al. [32] showed that trophoblasts contribute to maternal–fetal tolerance by secreting IL-35, which inhibits proliferation and induces the conversion of metaphase Tconv cells to iTr35 at the maternal–fetal interface. Further studies found that the reduction of IL-35 in trophoblasts and the resulting reduction in metaphase iTr35 are important causes of spontaneous abortion [32]. These results provide important clues to the pathogenesis of abnormal pregnancies.

### iTr35 cells and myelodysplastic syndromes

Myelodysplastic syndrome is one of the common malignant hematological diseases, and its relationship with the abnormal immune function of the organism has attracted much attention. A study found that the expression level of IL-35 and the proportion of iTr35 cells were positively correlated, and the proportion of iTr35 cells increased significantly with the increase in disease risk classification [33]. These findings suggest that the imbalance in the number and function of IL-35 and iTr35 cells plays an important role in the pathogenesis of myelodysplastic syndromes and provides new targets for the immunotherapy of myelodysplastic syndromes.

### iTr35 cells and acute b-precursor lymphocytic leukemia

Malignant cloning of acute lymphocytic leukemia cells is closely associated with immune escape due to low levels of anti-tumor immunity in the body, but the exact mechanism is unclear. The study [34] found that the level of the iTr35 subpopulation was significantly higher in the peripheral blood of patients with acute B-precursor lymphocytic leukemia than that of healthy controls. The level of IL-35 was also significantly higher in patients with acute B-precursor lymphocytic leukemia, suggesting that iTr35 and IL-35 may be involved in the immune escape process of tumor cells and may be used as a new target for the immunotherapy of acute B-precursor lymphocytic leukemia.

### iTr35 cells and acute myeloid leukemia

Treg-mediated tumor immune escape is a key factor in the pathogenesis of acute myeloid leukemia. Tao et al. [35] found that iTr35 levels were elevated in patients with acute myeloid leukemia, and the levels of IL-35 were significantly elevated. iTr35 was high in the peripheral plasma of adult patients with acute myeloid leukemia and was significantly correlated with the clinical stage of malignancy. The results suggest that iTr35 is one of the important cellular sources of IL-35, which is involved in the immune escape process of tumor cells and may serve as a new therapeutic target for AML.

### iTr35 cells and colorectal cancer

Colorectal cancer is a major public health problem in China, whose underlying mechanisms are not fully understood. Several studies have suggested that an increased number of Treg cells in the blood and tumor may contribute to a state of impaired immunity to colorectal cancer, and these studies suggest that strategies to overcome Treg cell activity may be beneficial in the treatment of human colorectal cancer [36–38]. Ma et al. [39] found that elevated IL-35 induced the production of iTr35 cells, inhibited the proliferation of effector T cells, and activated STAT1/STAT3 heterodimers in human CD4 + T cells. These results may support the potential therapeutic role of IL-35 in colorectal cancer treatment.

### iTr35 cells and breast cancer

Breast cancer is the second leading cause of cancer-related deaths in Chinese women. Despite improvements in surgical and pharmacological strategies, breast cancer continues to have high rates of recurrence and metastasis, the main cause of which is immunosuppression in cancer [40]. Treg cells have been found to play a key role in maintaining a suppressive tumor microenvironment, thereby promoting tumor progression [41, 42]. Hao et al. [43] found that breast cancer cells also expressed and secreted IL-35 and that higher levels of IL-35 in breast cancer cells were closely associated with poor prognosis for breast cancer. They further found that breast cancer cell-derived IL-35 promoted the secretion of the inhibitory cytokine IL-10 and significantly decreased the secretion of the Th17-type cytokine IL-17 and the Th1-type cytokine IFN-γ in Tconv cells, which induced iTr35 by activating the transcription factors STAT1/STAT3 [43]. Recently, it has been found that IL-35 was also expressed in other types of cancer cells, such as pancreatic, colon, and hepatocellular carcinomas [44–46]. In vivo studies have found that IL-35 neutralizes and limits tumor growth in a variety of murine tumor models [47]. These studies provide evidence for a novel mechanism of tumor escape in cancer and suggest that IL-35 may be a novel biomarker and potential therapeutic target for tumors.

### iTr35 cells and atherosclerosis

Atherosclerosis has been widely recognized as a slow-progressing inflammatory disease. A growing body of evidence suggests that IL-35 could be an attractive target for future anti-atherosclerotic therapy due to its multiple atheroprotective properties. A study found that mice with reduced IL-35 by injection of neutralizing anti-EBI3 antibodies developed more severe aortic plaques [48]. First, the immunosuppressive and anti-inflammatory activity of IL-35 may be beneficial in vasculitis. Second, IL-35 can suppress dendritic cells and a variety of T cells, including pro-inflammatory cells Th1 and Th17. Third, IL-35 supports the proliferation of Treg cells, enhances their suppressive function, and induces their differentiation to iTr35. Fourth, IL-35 promotes the production of the anti-inflammatory cytokine IL-10 and downregulates the expression of the pro-inflammatory factor IL-17. Finally, IL-35 is inducible, a fact that may inform the development of new effective strategies for the treatment of atherosclerosis [49].

### iTr35 cells and fibrotic diseases

Fibrosis complicates many diseases and leads to serious consequences in the lungs, liver, heart, kidneys, and skin. In essence, fibrosis is caused by excessive, persistent, and often irreversible aggregation of extracellular matrix or collagen during tissue damage and repair. Recent studies have shown that the pathology of fibrosis, particularly lung and liver fibrosis, involves various types of immune cells and soluble mediators, including IL-35. In addition, IL-35 has been reported to inhibit fibrotic disease. As reported, induction of immune tolerance by mucosal inoculation with type V collagen attenuated atherosclerosis in mice [48]. In addition, they found that IL-35-secreting iTr35 cells may play an important role in establishing and maintaining immune tolerance to collagen [50]. These results indicate that IL-35 plays a role in collagen deposition. Idiopathic pulmonary fibrosis is the most common clinical of idiopathic interstitial pneumonia, and its incidence is on the rise. Study [51] found that IL-35 and iTr35 levels were reduced in the peripheral blood of patients with idiopathic pulmonary fibrosis compared to healthy controls, and their levels were correlated with disease severity, suggesting that IL-35 and iTr35 cells are involved in the pathogenesis of idiopathic pulmonary fibrosis.

## Summary and outlook

In summary, iTr35 cells may have potential clinical value to serve as a biomarker and a new therapeutic target for many inflammatory diseases. Future in-depth studies are needed to explore their mechanisms of action in autoimmune diseases.

## Data Availability

All data included in this study are available upon request by contact with the corresponding author.
